# Influencia de la farmacogenética en la diversidad de respuesta a las estatinas asociada a las reacciones adversas

**DOI:** 10.1515/almed-2023-0064

**Published:** 2023-10-05

**Authors:** Jaime I. Sainz de Medrano Sainz, Mercè Brunet Serra

**Affiliations:** Servicio de Bioquímica y Genética Molecular, Centro de Diagnóstico Biomédico, Hospital Clínic de Barcelona, Barcelona, España; Jefa de sección de Farmacología y Toxicología, Servicio de Bioquímica y Genética Molecular, Centro de Diagnóstico Biomédico, Hospital Clínic de Barcelona, Barcelona, España

**Keywords:** estatinas, farmacogenética, reacciones adversas, *SLCO1B1*, medicina de precisión

## Abstract

**Introducción:**

Las estatinas son unos de los medicamentos más prescritos en los países desarrollados por ser el tratamiento de elección para reducir los niveles de colesterol ayudando así a prevenir la enfermedad cardiovascular. Sin embargo, un gran número de pacientes sufre reacciones adversas, en especial miotoxicidad. Entre los factores que influyen en la diversidad de respuesta, la farmacogenética puede jugar un papel relevante especialmente en la prevención de los efectos adversos asociados a estos medicamentos.

**Contenido:**

Revisión de los conocimientos actuales sobre la influencia de la farmacogenética en la aparición y prevención de las reacciones adversas asociadas a estatinas, así como del beneficio clínico del test farmacogenético anticipado.

**Resumen:**

Variaciones genéticas en *SLCO1B1* (rs4149056) para todas las estatinas; en *ABCG2* (rs2231142) para rosuvastatina; o en *CYP2C9* (rs1799853 y rs1057910) para fluvastatina están asociadas a un incremento de las reacciones adversas de tipo muscular y a una baja adherencia al tratamiento. Además, diversos fármacos inhibidores de estos transportadores y enzimas de biotransformación incrementan la exposición sistémica de las estatinas favoreciendo la aparición de las reacciones adversas.

**Perspectiva:**

La implementación clínica del análisis anticipado de este panel de farmacogenética evitaría en gran parte la aparición de reacciones adversas. Además, la estandarización en la identificación de los efectos adversos, en la metodología e interpretación del genotipo, permitirá obtener resultados más concluyentes sobre la asociación entre las variantes genéticas del *SLCO1B1, ABCG y CYP2C9* y la aparición de reacciones adversas y establecer recomendaciones para alcanzar tratamientos más personalizados para cada estatina.

## Introducción

La medicina de precisión, basada en la aplicación estandarizada de criterios clínicos, a menudo fundamentados en la interpretación de diversos biomarcadores válidos, permite la implementación de nuevas estrategias preventivas, diagnósticas y terapéuticas que consideran las características de cada paciente. Uno de sus objetivos es personalizar la prevención o el tratamiento farmacológico de una enfermedad teniendo en cuenta una serie de factores que evidencian la elevada variabilidad interindividual. Las diferencias entre las respuestas, en cuanto a tipo e intensidad, de los pacientes frente a los fármacos pueden deberse a distintas causas entre las que destacan los factores genéticos (farmacogenética), ambientales (epigenética), la adherencia al tratamiento, las interacciones fármaco-fármaco, la fisiopatología y el origen étnico [1, 2].

La farmacogenética juega un papel fundamental en la medicina personalizada, y tiene como objetivo principal prevenir la aparición de efectos adversos y mejorar la eficacia de los fármacos [3, 4]. Esta mejora del perfil de eficacia y seguridad tiene especial interés en el contexto de pacientes polimedicamentados en los que la incidencia de reacciones adversas a los medicamentos (RAMs) y el fracaso terapéutico puede ser mayor.

La personalización de la prevención y del tratamiento farmacológico de las enfermedades cardiovasculares es de gran importancia debido a que son la principal causa de morbilidad y mortalidad en países desarrollados, representando un total de 874.613 muertes anuales en Estados Unidos y 4.1 millones en Europa, según datos de 2019, y 19.05 millones a nivel mundial en 2020 [5, 6]. En el caso de Europa esta incidencia equivale alrededor del 40 % de causas de muerte [6].

Se sabe que las lipoproteínas que contienen apo-B100, especialmente el colesterol de lipoproteínas de baja densidad (cLDL), son las principales causantes de la aterogénesis [7]. Además, la hipercolesterolemia es el principal objetivo de los programas de reducción de riesgo cardiovascular en los cuales el tratamiento con estatinas en prevención primaria puede reducir un 15 % el riesgo por muerte vascular por cada 38.6 mg/dL de cLDL reducido [6]. Diversas entidades académicas y científicas (American Heart Association (AHA), European Atherosclerosis Society (EAS)) a través de sus recomendaciones enfatizan la importancia del uso de estatinas en el tratamiento y la prevención de la enfermedad cardiovascular [8]. En 2018, la atorvastatina y la simvastatina fueron los fármacos más prescritos en Estados Unidos, número 1 y 10 respectivamente, donde 1 de cada 4 americanos de 40 años o más recibía tratamiento con estatinas [9]. En España, según datos de la Agencia Española de Medicamentos y Productos Sanitarios (AEMPS), la dosis diaria definida por 1,000 habitantes y día fue de 110 en 2021 siendo la dosis diaria de atorvastatina 63.34 y la de simvastatina 26.67 [10].

El colesterol se sintetiza a partir de acetil coenzima A. El paso limitante en su síntesis es la reducción del hidroximetilglutaril (HMG) a mevalonato a través de la enzima HMG-CoA reductasa. Akira Endo estableció la hipótesis de que algunos organismos inhibirían esta enzima como mecanismo de defensa ante microbios que necesitasen el colesterol para sobrevivir [7, 11]. Fue así como la Mevastatina, aislada del hongo *Penicillium citrinum* en 1970, con una estructura similar a la HMG-CoA y potente inhibidor competitivo de la HMG-CoA reductasa, se utilizó como el primer agente hipolipemiante [12]. Posteriormente, se desarrollaron otras estatinas [11]. Las de primera generación, lovastatina, pravastatina y fluvastatina, fueron introducidas en Estados Unidos a finales de los años 1980 y 1990, siendo las de más baja potencia. Las de segunda generación, atorvastatina y simvastatina, mejoraron la eficacia para reducir el cLDL. En cuanto a las de tercera generación, rosuvastatina demostró ser la más potente [13]. Aunque todas las estatinas tienen el mismo grupo farmacóforo, se diferencian entre ellas en el anillo que va unido al resto activo y que determinará su estructura química, la farmacocinética, el efecto clínico o las propiedades farmacológicas incluyendo la solubilidad. Ello explica que dispongamos de moléculas más hidrófilas, como la pravastatina y la rosuvastatina, y otras más lipófilas como la atorvastatina, la lovastatina, la fluvastatina, la pitavastatina y la simvastatina. Aparentemente las lipófilas tienen una mayor prevalencia de síntomas musculares asociados a estatinas (SAMS), lo que podría ser debido a que atraviesan pasivamente las membranas celulares en tejidos musculares y otros extrahepáticos, aunque se requieren más estudios para llegar a establecer esta conclusión [14].

A pesar de ser un grupo farmacológico ampliamente utilizado, hay un número considerable de pacientes que sufren RAMs [15]. Los SAMS son los más informados y los que llevan a una baja adherencia al tratamiento e incluso a su discontinuación, lo que conlleva un aumento del cLDL y del riesgo de enfermedad cardiovascular [8, 9, 12, 14, 16].

La asociación entre la farmacogenética de las estatinas, administradas para el tratamiento preventivo de las enfermedades cardiovasculares, y la aparición de efectos adversos es bien conocida y puede variar según el tipo y dosis de estatina [17]. El *Clinical Pharmacogenetics Implementation Consortium* (CPIC) publicó una guía de actualización en 2022 [9] recomendando el análisis de los genotipos d*e SLCO1B1, ABCG2* y *CYP2C9* por su elevada asociación con el aumento de la exposición sistémica de las estatinas y el consiguiente incremento del riesgo de SAMS (evidencia 1A).

La farmacogenética juega un papel fundamental en la medicina personalizada por lo que su implementación en rutina clínica es crucial para elegir la estatina y la dosis más adecuada para cada paciente. Esta revisión se centra en la influencia de la farmacogenética en la diversidad de respuesta a estos fármacos y en su potencial para prevenir las reacciones adversas. Para llevarla a cabo se ha hecho una búsqueda bibliográfica en PubMed, desde el año 2015 hasta el 2023 (incluye también algunos artículos de referencia previos) utilizando como palabras clave: atorvastatina, simvastatina, rosuvastatina, pitavastatina, estatinas, farmacogenética, *SLCO1B1, ABCG2, CYP2C9, CYP3A4*, miopatía, mialgias, rabdomiólisis, reacciones adversas, medicina de precisión, metaanálisis. Se han considerado de forma especial las recomendaciones procedentes de las principales guías de farmacogenética CPIC, The Dutch Pharmacogenetics Working Group (DPWG) y de la web Pharmacogenomics Knowledgebase (PharmGKB).

## Farmacología de las estatinas

Como ya hemos mencionado, si bien las estatinas comparten una estructura química básica similar presentan una serie de diferencias entre ellas que determinarán la diversidad en sus propiedades farmacológicas [13, 14, 18].

### Farmacodinamia: mecanismo de acción y reacciones adversas

#### Mecanismo de acción

**Figura 1:**
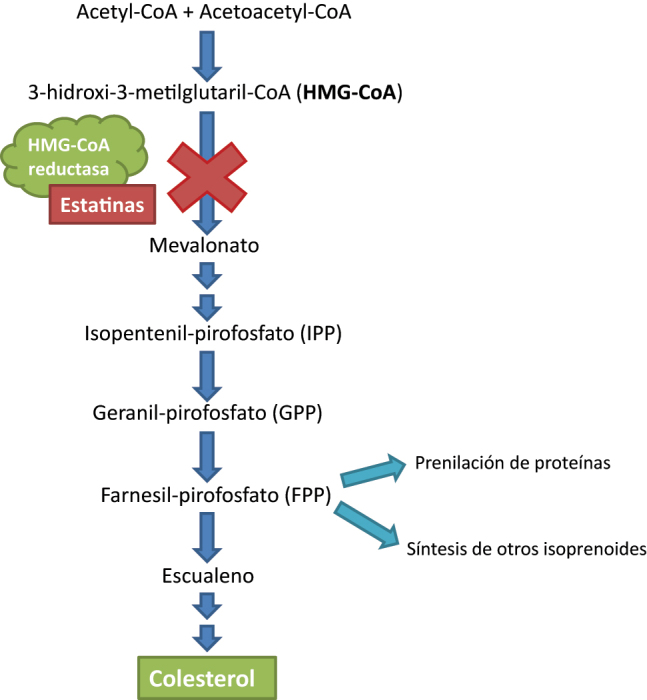
Mecanismo de acción de las estatinas actuando en la vía del mevalonato. Adaptado de [11].

Las estatinas reducen la síntesis del colesterol en el hígado inhibiendo de forma competitiva la enzima HMG-CoA reductasa (Figura 1) [11, 19] paso limitante en la síntesis. La reducción de los niveles intracelulares de colesterol provoca un aumento de receptores LDL (LDLR) en el hepatocito favoreciendo una mayor captación y, como consecuencia, una reducción en la concentración plasmática de LDL y otras lipoproteínas que contienen ApoB.

En cuanto al nivel de reducción del colesterol con lipoproteína de baja densidad (cLDL), es dosis dependiente y varía según la estatina. Las de alta intensidad (atorvastatina 40/80 mg y rosuvastatina 20/40 mg) reducen, de media, el cLDL un ≥50 %, las de moderada intensidad (atorvastatina 10/20 mg, rosuvastatina 5/10 mg, simvastatina 20/40 mg, pravastatina 40/80 mg, lovastatina 40/80 mg, fluvastatina 40/80 mg y pitavastatina 1/4 mg) entre un 30–49 % y las de baja intensidad un <30 % (simvastatina 10 mg, pravastatina 10/20 mg, lovastatina 20 mg y fluvastatina 20/40 mg). También reducen los niveles de triglicéridos entre un 10–20 %. El mecanismo no es del todo conocido, pero podría deberse a una mayor captación del colesterol con lipoproteínas de muy baja densidad (VLDL) en los hepatocitos, así como a una disminución en la producción de VLDL. Además, dependiendo de la dosis y de la estatina, aumentan el colesterol con lipoproteínas de alta densidad (HDL) entre 1–10 % [20]. En cuanto al efecto sobre la lipoproteína (a), las estatinas solo afectan ligeramente a sus niveles plasmáticos y respecto a los efectos pleiotrópicos, como el efecto antiinflamatorio y antioxidante, han sido evidenciados *in vitro* pero su relevancia clínica no está demostrada [21, 22].

Las diferencias en el grado de unión entre las distintas estatinas y la HMG-CoA reductasa podría dar lugar a diferencias en la potencia de acción. Así, atorvastatina y rosuvastatina tienen un enlace de hidrógeno extra en la unión con la enzima y esta última además también interacciona mediante enlaces polares, lo que podría explicar su mayor potencia en el efecto hipolipemiante [19].

#### Reacciones adversas: SAMS

Las estatinas inhiben la síntesis del colesterol en el hígado, pero en aquellas situaciones en las que sus concentraciones plasmáticas son elevadas también lo hacen en otros tejidos extrahepáticos, incluyendo el tejido muscular, donde pueden llegan a provocar el 95 % de los efectos adversos. Aunque esta asociación y el mecanismo de acción no están del todo descritos, se estima que puede afectar hasta un 30 % de los pacientes [14]. Estos síntomas varían desde una forma leve a enfermedad muscular llegando incluso a rabdomiólisis potencialmente letal [17, 23].

El porcentaje de SAMS es difícil de cuantificar debido a que está basado en el autodiagnóstico de los pacientes, a veces el dolor es pasajero, se resuelve solo y puede ser causado por muchos factores [14, 16]. Esto unido a una falta de estandarización donde se usan indistintamente términos como mialgia, miositis o miopatía hace difícil la comparación entre los resultados de diferentes estudios. Con más detalle, resultados procedentes de una revisión de estudios clínicos aleatorizados en pacientes sin enfermedad cardiovascular, tratados con estatinas, demuestran la asociación entre la aparición de síntomas musculares (principalmente debilidad o rigidez) y las estatinas, sin que ello represente una clara alteración clínica muscular de tipo mialgia, miopatía, o rabdomiólisis [17]. Por otra parte, un estudio doble ciego con 12.064 pacientes demostró que la miopatía ocurría en un 0.03 % de los pacientes con bajas dosis de simvastatina y en un 0.9 % con simvastatina de 80 mg [14]. En consonancia con estos resultados, Stillemans y col [24]. demuestran la influencia de la dosis y exposición de la atorvastatina con el riesgo de aparición de mialgias. En controversia con estos resultados, un metaanálisis de Irwin JC y col., que incluía un total de 192.977 pacientes, indicó que había un ligero aumento de SAMS en el grupo de pacientes tratados con estatinas, pero no pudo establecer una asociación dosis-dependiente [25]. Por último, un estudio en pacientes de edad avanzada, un metaanálisis que incluyó 18.192 participantes tratados con: atorvastatina, fluvastatina, lovastatina, pravastatina o rosuvastatina evidenció que no había diferencias en cuanto a reacciones adversas en el grupo tratado en comparación con el placebo [26]. Todos estos resultados evidencian un cierto grado de inconsistencia en cuanto a la asociación entre las estatinas y la aparición de efectos adversos clínicos musculares.

Para solucionar este problema el consorcio PREDICTION-ADR ha querido clasificar el fenotipo y estandarizar la nomenclatura del daño muscular estableciendo una escala desde SRM0 (miotoxicidad relacionada con estatinas) para los casos más leves a SRM6 para los más graves [27].

Obviamente, la importancia del dolor muscular radica en que es una de las principales causas de una baja adherencia o incluso para dejar el tratamiento [27]. Diversos estudios exponen que la discontinuación de las estatinas tras 6 meses de tratamiento puede afectar al 50 % de los pacientes tratados [7, 28]. Según la guía CPIC los SAMS ocurren en 1–7 % de los pacientes y el riesgo es aproximadamente 6 veces mayor en pacientes tratados con altas dosis en comparación a los que reciben dosis bajas [9]. Por todo ello, además de ser imprescindible la estandarización de la identificación y registro de las reacciones adversas musculares, como propone el consorcio PREDICTION-ADR, sería de gran beneficio clínico disponer de biomarcadores específicos para estos efectos adversos.

El uso de potenciales biomarcadores predictivos de daño muscular como la creatina quinasa (CK), con resultados controvertidos [16], o más recientemente algunos miRNAs, el miR-145 [29] o miR-499-5 [30], sería de especial ayuda para poder identificar de forma específica estos efectos adversos asociados a las estatinas.

En cuanto a otros efectos adversos, a nivel hepático se ha observado una leve elevación de la alanina aminotransferasa (ALT) en un 0.5–2 % de los pacientes tratados con estatinas de alta intensidad o altas dosis sin que ello se asocie con hepatotoxicidad [22]. Además, varios estudios han demostrado un aumento de Diabetes Mellitus tipo 2 sobre todo con altas dosis de estatinas y en pacientes ancianos con sobrepeso o con resistencia a la insulina [22].

La estandarización de la identificación y registro de las SAMS u otros efectos adversos asociados a estatinas, así como el desarrollo de biomarcadores específicos para estas RAMs permitirán evaluar una clara asociación entre tipo de estatina, dosis y aparición de efectos adversos. Además, los estudios farmacogenéticos posibilitan la identificación de aquellos pacientes con mayor riesgo de desarrollar SAMS (ver apartado 3. Farmacogenética). En cualquier caso, es de destacar que estos efectos adversos son moderados comparados con el beneficio clínico que conlleva el tratamiento con estatinas para la prevención de las enfermedades cardiovasculares [17, 22].

### Farmacocinética e interacciones medicamentosas

Las estatinas se administran por vía oral y su estructura química determina su solubilidad, con gran influencia en la absorción, distribución, metabolismo y excreción de cada una de ellas. En la Tabla 1 se exponen las características farmacocinéticas de las estatinas más utilizadas en nuestro medio [11, 12, 19, 31].

**Tabla 1:** Farmacocinética de las estatinas.

	Potencia, nM^a^	Absorción oral, %	Biodisponibilidad, %	Extracción hepática, %	Unión a proteínas, %	t1/2, h	Vd, L/kg	Metabolismo CYP450	Excreción renal, %
Atorvastatina	1,16	30	12	70	>98	7–20	5,4	3A4 (2C8)^b^	<5
Simvastatina	1–2	60–85	<5	>80	>95	2–5	–	3A4 (2C8, 2D6)^b^	13
Rosuvastatina	0,16	50	20	63	90	20	1,7	Limitada	10
Pravastatina	4	35	18	45	50	1–3	0,46	Limitada	20
Lovastatina	2–4	30	5	>70	>98	2–5	–	3A4	10
Fluvastatina	3–10	98	30	>70	>98	1–3	0,42	2C9	6
Pitavastatina	0,1	80	60	?	96	10–13	0,70	Limitada	–

^a^Medido como IC50 (concentración para inhibir un 50 %). ^b^Metabolismo minoritario. Adaptado de [11, 12, 19, 31]. CYP, citocromo P; t1/2, semivida de eliminación; Vd, volumen de distribución.

Lovastatina, simvastatina y pravastatina derivan de metabolitos fúngicos mientras que el resto son sintéticas. La biodisponibilidad varía desde un 5 % para simvastatina y lovastatina hasta un 60 % para pitavastatina. En la mayoría de estos agentes la biodisponibilidad es relativamente baja debido a un alto efecto del primer paso hepático que por otro lado favorece la acción farmacológica de las estatinas en este órgano [19].

En cuanto a la solubilidad, atorvastatina, simvastatina, lovastatina, fluvastatina y pitavastatina son relativamente lipófilas por lo que serán transportadas por difusión pasiva, metabolizadas por el citocromo P450 y eliminadas por vía biliar [19]. El complejo CYP3A4 metaboliza la mayoría de los fármacos incluyendo lovastatina, simvastatina y atorvastatina, mientras que fluvastatina es metabolizada principalmente por CYP2C9. Respecto a las más hidrófilas, rosuvastatina y pravastatina, requieren transporte activo en el hígado, no son metabolizadas de forma significativa por CYP450 y son excretadas por vía hepática y renal [16, 19].

**Figura 2:**
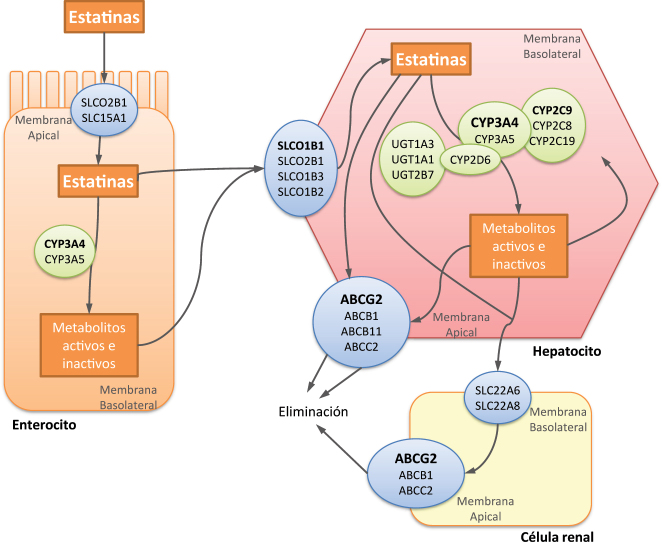
Influencia de la farmacogenética en la distribución, exposición y efecto de las estatinas. Figura adaptada de [8, 32].

La incidencia de miopatía por estatinas es baja en monoterapia. Sin embargo, aumenta al coadministrarlas con otros fármacos que alteran su farmacocinética [9]. De hecho, se ha estimado que un 60 % de los casos de rabdomiólisis por estatinas se deben a interacciones con otros fármacos [16]. Las estatinas son usadas frecuentemente en combinación con otros fármacos debido a que muchos pacientes con hiperlipidemia padecen otras enfermedades como diabetes o hipertensión. El nivel de interacción entre fármacos está influenciado tanto por el grado de metabolización de cada estatina por el citocromo P450 así como por la afinidad de las mismas con los transportadores de membrana, como el transportador de aniones orgánicos 1B1 (OATP1B1) o la proteína de resistencia al cáncer de mama (BCRP) [16, 31]. En la Figura 2 y en la Tabla Suplementaria 1 se reflejan las proteínas reguladoras de transporte y los enzimas de biotransformación que influyen en la distribución y exposición de las estatinas [9, 32]. Todas ellas sufren metabolismo hepático por enzimas CYP, siendo minoritario para pravastatina, rosuvastatina y pitavastatina. La isoenzima CYP3A4 es la principal involucrada pero también participan otras como CYP2C8, CYP2C9, CYP2C19 y CYP2D6. Por lo tanto, todos los fármacos que interaccionen tanto con estas enzimas de biotransformación como con las proteínas transportadoras podrán aumentar el riesgo de aparición de efectos adversos [14, 21, 22]. La mayoría de estos fármacos están recogidos en la Tabla 2.

**Tabla 2:** Inhibidores e inductores de enzimas de biotransformación y proteínas transportadoras que son sustratos de estatinas.

Enzima o proteína transportadora	Estatina	Inhibidor	Inductor
CYP2C9	Fluvastatina, rosuvastatina (CYP2C19)^a^	Amiodarona, capecitabina, etravirina, fluconazol, fluvoxamina, fluvastatina, ketoconazol, metronidazol, miconazol, oxandrolona, sulfametoxazol/trimetoprima, voriconazol, zafirlukast	Carbamazepina, fenobarbital, fenitoina, rifampicina
CYP3A4	Atorvastatina, lovastatina, simvastatina	Amiodarona, amlodipino, aprepitant, atorvastatina, bicalutamida, cilostazol, cimetidina, ciprofloxacino, claritromicina, conivaptan, ciclosporina, diltiazem, eritromicina, fluconazol, fluoxetina, fluvoxamina, zumo de pomelo, imatinib, isoniazida, itraconazol, ketoconazol, mibefradil, midazolam, nefazodona, nilotinib, posaconazol, inhibidores de proteasa, ranolazina, sertralina, tacrolimus, telitromicina, ticagrelor, antidepresivos tricíclicos, verapamilo, voriconazol	Aprepitant, bosentan, carbamazepina, ciclofosfamida, corticoides, efavirenz, modafinilo, nafcilina, nevirapina, fenitoína, pioglitazona, fenobarbital, rifampicina, hierba de San Juan
P-gp (*ABCB1*)^b^	Atorvastatina, lovastatina, pitavastatina, simvastatina	Amiodarona, atorvastatina, azitromicina, captopril, carvedilol, cimetidina, claritromicina, colchicina, conivaptan, ciclosporina, diltiazem, dipiridamol, dronedarona, eritromicina, felodipina, zumo de pomelo, itraconazol, ketoconazol, lovastatina, mefloquina, nicardipino, omeprazol, inhbidores de proteasa, quinidina, ranolazina, reserpina, darolutamida sertralina, simvastatina, tacrolimus, verapamilo	Carbamacepina, fenitoína, rifampicina, hierba de San Juan
BCRP (*ABCG2*)^b^	Rosuvastatina	Darolutamida	
OATP1B1 (*SLCO1B1*)^b^	Atorvastatina, pitavastatina, pravastatina, rosuvastatina, simvastatina	Carbamacepina, claritromicina, ciclosporina, eritromicina, gemfibrozilo, Inhibidores de proteasa, roxitromicina, rifampicina, sildenafilo, sacubitril, telitromicina, glecaprevir, pibrentasvir	
OATP1B3 (*SLCO1B3*)^b^	Fluvastatina, pravastatina, rosuvastatina	Claritromicina, ciclosporina, eritromicina, rifampicina, roxitromicina, rifampicina, sacubitril, telitromicina, glecaprevir, pibrentasvir	

^a^Metabolismo minoritario. ^b^Gen codificante. Adaptado [8, 16, 31]. CYP, citocromo P; OATP, transportador de aniones orgánicos; P-gp, glicoproteína-P.

Como ejemplo de fármaco lipófilo, la atorvastatina es metabolizada en un 85 % mediante la enzima CYP3A4 y además es sustrato de OATP1B1, BCRP y también de transportadores de flujo dependientes de ATP como la proteína de resistencia a múltiples fármacos (MDR1) [9, 16]. La expresión y actividad de estas proteínas reguladoras de transporte son un factor limitante para el efecto fisiológico de las estatinas ya que determinarán la concentración de fármaco que entrará o que será expulsado del hepatocito. Por lo tanto, y como veremos en el apartado de farmacogenética, variantes genéticas de los genes que codificarán estas proteínas estarán asociadas a cambios en la concentración del fármaco y a su efecto.

Se han descrito muchas interacciones entre la atorvastatina y otros fármacos que son potentes inhibidores del CYP3A4, como por ejemplo: los azoles antifúngicos (itraconazol, voriconazol); los macrólidos (eritromicina y claritromicina); los inhibidores de la proteasa del HIV (darunavir, fosamprenavir, ritonavir, saquinavir y tipranavir) y del HCV (telaprevir) y los antagonistas del canal de calcio (mibefradil), que conllevan a un incremento considerable del área bajo la curva (AUC) de la concentración plasmática de la atorvastatina.

Un estudio sobre las interacciones del faldaprevir ha puesto de manifiesto que este fármaco aumenta unas 8 veces la exposición sistémica de la atorvastatina, pero la vida media de eliminación de esta última se reduce ligeramente sugiriendo que esta interacción es principalmente debida a una inhibición del transportador de captación hepática OATP1B [33]. En línea con este hallazgo, los resultados de los estudios de Yamazaki y col [34]. y los de Alam K y col [35]. demuestran que la inhibición de este transportador SLCO1B1 por el isavuconazol y la cloroquina, respectivamente, favorece un incremento en la exposición y en el riesgo de SAMS.

La pravastatina, a diferencia de la atorvastatina, es un compuesto hidrofílico y no es metabolizado significativamente por las enzimas CYP, por lo que los potentes inhibidores o inductores de CYP3A4, CYP2C9 o CYP2C19 no afectarán significativamente a la farmacocinética de la pravastatina, lo que la convierte en una de las estatinas de elección en pacientes polimedicamentados. Además, el hecho de tener un carácter hidrófilo evita que penetre a través de las membranas celulares de otros tejidos como el muscular [19].

En cuanto a los transportadores OATP2B1 y BCRP, están expresados mayoritariamente en los enterocitos por lo que pueden promover o atenuar la absorción, respectivamente. Diversos estudios han demostrado que el tratamiento concomitante de pravastatina con fármacos inhibidores del OATP1B1 como la ciclosporina, el glecaprevir o el pibrentasvir aumentan de forma significativa el valor del AUC de la pravastatina en plasma [14, 36].

Respecto a nuevos biomarcadores, un estudio sobre la interacción entre rosuvastatina y rifampicina demostró que la coproporfirina podría ser utilizada como biomarcador endógeno que refleje la inhibición de OATP1B. El desarrollo e implementación de biomarcadores que demuestren el grado de inhibición de los transportadores es importante en la evaluación de su influencia sobre el efecto de las estatinas [37].

## Farmacogenética

Para poder implementar la farmacogenética en la práctica clínica, el grado de evidencia en la asociación gen-fármaco (gen accionable que permite recomendaciones sobre el tratamiento) establecido en las distintas guías debe ser el más elevado (1A). Entre las guías más importantes, cabe destacar el CPIC, DPWG, PharmGKB, The Canadian Pharmacogenomics Network for Drug Safety (CPNDS) o The French National Network of Pharmacogenetics (RNPGx). Las metodologías para calificar la evidencia científica, las recomendaciones terapéuticas segun genotipado y el grado de recomendación varían según la guía [38, 39]. Sin embargo, hay trece genes seleccionados en común por CPIC, DPWG y PharmGKB, de los cuales 3 tienen efecto sobre las estatinas: *ABCG2, CYP2C9 y SLCO1B1*. Actualmente, las definiciones de los alelos del *SLCO1B1* están en consonancia con los estándares de la PharmVar [40].

El beneficio de hacer test farmacogenéticos antes de empezar el tratamiento está bien documentado por las agencias reguladoras, como la *Food and Drug Administration* (FDA) o la *European Medicines Agency* (EMA), que están a favor de realizar test genéticos antes de empezar el tratamiento de ciertos fármacos. Sin embargo, su implementación en la práctica clínica tardó en desarrollarse debido a que las distintas entidades trabajaban sobre distintos fármacos, genes o variantes genéticas por gen [39]. Por este motivo diversos países europeos han seleccionado un panel farmacogenético que incluye 12 genes, 58 variantes genéticas a analizar que permiten establecer recomendaciones sobre 57 fármacos, con grado de evidencia 1A según las guías CPIC y DPWG [41].

Este panel se ha evaluado en un estudio multicéntrico europeo y los resultados demuestran que la terapia individualizada basada en test farmacogenéticos, según las combinaciones específicas fármaco-gen, permite disminuir los efectos adversos y mejorar la evolución clínica de los pacientes [42]. En febrero de 2023 el Consorcio Farmacogenómico Ubicuo (U-PGx) publicó los resultados del estudio multicéntrico, elaborado en 7 países europeos con un total de 6.944 pacientes, llamado test farmacogenético preventivo (o anticipado) para prevenir reacciones adversas (PREPARE). El objetivo era evaluar los beneficios de realizar el test preventivo del panel farmacogenético previamente seleccionado [41], conocido como “pasaporte genético”. Este panel incluye el genotipo del *CYP2C9* y *SLCO1B1*. Es de destacar que la atorvastatina fue el fármaco más evaluado y los resultados evidenciaron una reducción del 30 % en las reacciones adversas clínicamente relevantes para los fármacos evaluados, demostrando además poder hacerlo de forma costo-efectiva [42].

En los últimos años se han llevado a cabo diversos estudios para valorar el impacto de las variaciones genéticas de enzimas de biotransformación y transportadores en la farmacocinética y en la farmacodinamia de las estatinas. Debido a que en la mayoría de estudios la relación dosis-respuesta ha sido observada en la aparición de toxicidad con un nivel de evidencia 1A (mientras que referente a la eficacia el grado de evidencia es menor), los polimorfismos que afecten a la farmacocinética de las estatinas podrán influir tanto en la aparición como en la gravedad de las reacciones adversas [8, 9, 14, 16]. En la guía del CPIC actualizada en el año 2022 [9] se expone la influencia de la farmacogenética en el fenotipo de las estatinas incluyendo farmacocinética, SAMS, hepatotoxicidad, efecto hipolipemiante y eficacia clínica. En esta guía se consideran los estudios más relevantes así como la opinión de expertos para evaluar varios genes y los que tuvieron mayor nivel de evidencia en su asociación con la aparición de las RAM fueron *SLCO1B1* (todas las estatinas), *ABCG2* (rosuvastatina) y *CYP2C9* (fluvastatina), estableciéndose una serie de recomendaciones que podrían ayudar a reducir los SAMS. Aunque hay revisiones acerca de otras acciones como la influencia en el efecto hipolipemiante, la guía solo establece recomendaciones acerca de las reacciones adversas. En cuanto a otros genes como *HMGCR*, *CYP3A4* o *CYP3A5*, aunque se están realizando estudios, todavía no hay suficiente evidencia para implementarlos a la clínica. En la Tabla 3 se resume la relación entre el genotipo (diplotipos) y su asociación con la función de las proteínas reguladoras de transporte, o en su caso la predicción del fenotipo metabolizador para *SLCO1B1*, *ABCG2* y *CYP2C9,* respectivamente, basándonos en los documentos del CPIC [9].

**Tabla 3:** Predicción del probable fenotipo basado en el genotipo de *SLCO1B1, ABCG2 y CYP2C9*.

Gen	Fenotipo	Score	Genotipo	Ejemplos de diplotipos
*SLCO1B1*	Función aumentada	n/a	Portador de dos alelos de función aumentada	*14/*14
	Función normal	n/a	Portador de dos alelos de función normal o uno de función normal y uno de función aumentada	*1/*1, *1/*14
	Función disminuida	n/a	Portador de un alelo normal o de función aumentada y de un alelo afuncional	*1/*5, 1/*15 (c.521T>C rs4149056)
	Función ineficaz	n/a	Portador de dos alelos afuncionales	*5/*5, *5/*15, *15/*15 (c.521T>C rs4149056)
*ABCG2*	Función normal	n/a	Portador de dos alelos de función normal	c.421 C/C (rs2231142)
	Función disminuida	n/a	Portador de un alelo normal y de un alelo afuncional	c.421 C/A (rs2231142)
	Función ineficaz	n/a	Portador de dos alelos afuncionales	c.421 A/A (rs2231142)
*CYP2C9*	Metabolizador normal	2	Portador de dos alelos de función normal	*1/*1
	Metabolizador intermedio	1,5	Portador de un alelo de función normal y uno afuncional	*1/*2 c.430C>T (rs1799853)^a^
		1	Portador de un alelo de función normal y uno afuncional o de dos alelos de función disminuida	*1/*3, *2/*2 (c.430C>T rs1799853)^a^ (c.1075A>C rs1057910)^b^
	Metabolizador ineficaz	0,5	Portador de un alelo de función disminuida y de uno afuncional	*2/*3 (c.430C>T rs1799853)^a^ (c.1075A>C rs1057910)^b^
		0	Portador de dos alelos afuncionales	*3/*3 (c.1075A>C rs1057910)^b^

^a^Expresado en alelo 2. ^b^Expresado en alelo 3. Adaptado de [9]. n/a, no aplicable.

A continuación, se describen los tres genes mencionados y cómo su genotipado nos permite predecir el fenotipo metabolizador o de función transportadora y las recomendaciones para el tratamiento.

### SLCO1B1 (transportador de aniones orgánicos de la familia 1B1/OATP1B1 o OATP-C)

Facilita la captación hepática de estatinas (y de compuestos endógenos como bilirrubina o 17-beta-glucuronosilestradiol). Una función disminuida, ya sea heredada genéticamente o adquirida debido a fármacos inhibidores, puede aumentar la exposición sistémica generando SAMS. El gen *SLCO1B1* tiene 109kilobases, se localiza en el cromosoma 12 (Chr 12p12.2) y aunque se han identificado distintas variantes de nucleótido único (SNVs) solo algunas tienen relevancia clínica. La más común y la que tiene el nivel más alto de evidencia clínica es c.521T>C, rs4149056, presente en los alelos *5 o *15 y está asociada a un aumento de la exposición sistémica de la estatina y aparación de SAMS. Hay diferencias en la frecuencia alélica según el origen étnico, siendo en Europa de 0.02 para SLCO1B1*5 y 0.15 para SLCO1B1*15.

Las personas con dos alelos de función aumentada (SLCO1B1*14/*14) tienen un fenotipo de función aumentada. Aquellos con un alelo normal y uno de función aumentada (SLCO1B1*1/*14) o con dos alelos de función normal (SLCO1B1*1/*1) tienen un fenotipo de función normal. Por último, aquellos que tengan un alelo no funcional (p.ej., SLCO1B1*5) junto a un alelo de función normal o aumentada tienen un fenotipo de función disminuida y aquellos que tengan dos alelos no funcionales (p.ej., SLCO1B1*5/*5) tienen un fenotipo de función ineficaz [9, 43]. Según diversos estudios, las variantes en *SLCO1B1* han demostrado solo una pequeña disminución (<5 %) en el efecto hipolipemiante de simvastatina, atorvastatina, lovastatina y pravastatina. Un metaanálisis realizado en 2015 no ha encontrado diferencias significativas para *SLCO1B1* c521T>C excepto para la simvastatina donde tenía un mayor efecto [44]. Otro metaanálisis concluyó que la fluvastatina en pacientes *SLCO1B1* TT produjo a una mayor reducción de colesterol total y LDL [45]. En la misma línea se demostró que los pacientes TT tenían un mayor efecto hipolipemiante comparado con heterocigotos [44].

Por otro lado, varios estudios sí que han demostrado una clara evidencia respecto al riesgo de toxicidad al aumentar las concentraciones sistémicas de algunas estatinas e incrementar por tanto el riesgo de miopatía. En un estudio (n=59) en el que la terapia estaba guiada por el genotipado de SLCO1B1*5, los pacientes portadores mostraron un aumento de la reducción de C-LDL y una mejora en la adherencia en comparación con el grupo control [8].

### ABCG2 (transportador perteneciente a la superfamilia de transportadores ABC (ATP binding casette) conocido también como transportador BCRP)

Se expresa en hígado, intestino y barrera hematoencefálica. Facilita la exportación de compuestos al espacio extracelular y el alelo A está asociado con una disminución del 30–40 % de la proteína y con un aumento de los niveles plasmáticos de rosuvastatina. El gen *ABCG2* tiene 66kilobases, se localiza en el cromosoma 4 (Chr 4q22.1) y la variante más estudiada es c.421C>A (rs2231142). Su frecuencia alélica varía según la etnia siendo en Europa de 0.1 para el alelo variante.

Los pacientes portadores de un alelo de función normal y uno de función disminuida tienen un fenotipo de función disminuida mientras que aquellos portadores de dos alelos no funcionales tienen un fenotipo de función ineficaz [9]. Un metaanálisis que incluyó 423 pacientes demostró que los portadores del alelo A en *ABCG2* 421C>A tenían una concentración aumentada de rosuvastatina. Debido a que la frecuencia del alelo A en población asiática es elevada (0.29) la FDA recomienda reducir la dosis en estos pacientes [46].

### CYP2C9

El citocromo P450 2C9 interviene en el metabolismo de fase I de muchos fármacos: se han identificado al menos 71 variantes alélicas pero las más estudiadas son el alelo 2, CYP2C9*2 (c.430C>T; rs1799853), y el alelo 3, CYP2C9*3 (c.1075A>C rs1057910), asociados a una disminución del 30–40 % y del 80 % de la función, respectivamente, y que lleva a un aumento de la exposición sistémica de la fluvastatina. Las frecuencias alélicas en Europa para el alelo 2 son de 0.13 y para el 3 de 0.07.

Los pacientes portadores de dos alelos de función normal (CYP2C9*1/*1) tienen un fenotipo metabolizador normal. Aquellos que sean portadores de un alelo normal y uno de función disminuida (CYP2C9*1/*2) o uno no funcional (CYP2C9*1/*3) y aquellos portadores de dos alelos de función disminuida (CYP2C9*2/*2) tienen un fenotipo de función intermedia (metabolizadores intermedios). Por último, aquellos que son portadores de un alelo de función disminuida y uno no funcional (CYP2C9*2/*3) o dos alelos no funcionales (CYP2C9*3/*3) tienen un fenotipo de función ineficaz (metabolizadores lentos). Además, a estos alelos se les da un valor según su actividad desde 0 hasta 1. Los metabolizadores ineficaces tienen un score de 0 y 0.5, los intermedios de 1 y 1.5 y los normales de 2 [9].

Todos aquellos pacientes que sean portadores de una variante en alguno de estos tres genes, que se traduzca en un fenotipo de metabolizador ineficaz o de transportador ineficaz, tendrán mayor riesgo de exposición sistémica elevada a una determinada estatina y consecuentemente mayor riesgo de aparición de SAMS, requiriendo un ajuste de dosis o cambio de estatina.

En cuanto a otros polimosfirmos no evaluados por esta guía, un estudio (n=156) demostró una posible asociación entre CYP3A5*1 y la acumulación de atorvastatina, aunque estos resultados deben confirmarse en el contexto de futuros estudios con cohortes independientes [47].

En la Tabla 4 se describen las recomendaciones terapéuticas sobre el ajuste de dosis para las diversas estatinas en base al fenotipo, predicho previamente por el análisis del genotipo. Los datos se basan en la guía del CPIC [9] y los metaanálisis [45, 46, 48]. Es aconsejable realizar el análisis del genotipo antes de iniciar el tratamiento y así poder considerar las recomendaciones pertinentes respecto al tipo y dosis de estatina.

**Tabla 4:** Recomendaciones de dosis basadas según el fenotipo y la estatina.

Estatina	Fenotipo	Implicaciones ^a^	Recomendación de dosis	Nivel de recomendación^b^
Atorvastatina	SLCO1B1 de función disminuida (c.521T>C rs4149056)^c^	Aumento de riesgo de miopatía comparado con función normal	Riesgo de miopatía en dosis >40 mg. Considerar terapia combinada en estos casos.	Moderado
	SLCO1B1 de función ineficaz (c.521T>C rs4149056)^c^	Aumento de riesgo de miopatía comparado con función normal y disminuida	Riesgo de miopatía en dosis >20 mg. Considerar rosuvastatina o terapia combinada en estos casos.	Moderado
Fluvastatina	SLCO1B1 de función disminuida (c.521T>C rs4149056)^c^	Aumento de riesgo de miopatía comparado con función normal	Riesgo de miopatía en dosis >40 mg	Moderado
	SLCO1B1 de función ineficaz (c.521T>C rs4149056)^c^	Aumento de riesgo de miopatía comparado con función normal y disminuida	Prescribir <40 mg. Si es necesario >40 mg considerar cambio de estatina o terapia combinada	Moderado
	Metabolizador normal CYP2C9	Exposición normal	Prescribir según guías	Fuerte
	Metabolizador intermedio CYP2C9 (c.430C>T rs1799853)^c^ (c.1075A>C rs1057910)^c^	Aumento de riesgo de miopatía comparado con metabolizador normal	Prescribir <40 mg. Si es necesario >40 mg considerar cambio de estatina o terapia combinada	Moderado
	Metabolizador ineficaz CYP2C9 (c.430C>T rs1799853)^c^ (c.1075A>C rs1057910)^c^	Aumento de riesgo de miopatía comparado con metabolizador normal o intermedio	Prescribir <20 mg. Si es necesario >20 mg considerar cambio de estatina o terapia combinada	Moderado
Lovastatina	SLCO1B1 de función disminuida (c.521T>C rs4149056)^c^	Aumento de riesgo de miopatía comparado con función normal	Prescribir estatina alternativa o limitar dosis a <20 mg	Moderado
	SLCO1B1 de función ineficaz (c.521T>C rs4149056)^c^	Aumento de riesgo de miopatía comparado con función normal y disminuida	Prescribir estatina alternativa	Moderado
Pitavastatina	SLCO1B1 de función disminuida (c.521T>C rs4149056)^c^	Aumento de riesgo de miopatía comparado con función normal	Riesgo de miopatía en dosis >2 mg. Considerar cambio de estatina o terapia combinada en estos casos.	Moderado
	SLCO1B1 de función ineficaz (c.521T>C rs4149056)^c^	Aumento de riesgo de miopatía comparado con función normal y disminuida	Riesgo de miopatía en dosis >1 mg. Considerar cambio de estatina o terapia combinada en estos casos.	Moderado
Pravastatina	SLCO1B1 de función disminuida (c.521T>C rs4149056)^c^	Aumento de riesgo de miopatía comparado con función normal	Riesgo de miopatía en dosis >40 mg	Moderado
	SLCO1B1 de función ineficaz (c.521T>C rs4149056)^c^	Aumento de riesgo de miopatía comparado con función normal y disminuida	Prescribir <40 mg. Si es necesario >40 mg considerar cambio de estatina o terapia combinada	Moderado
Rosuvastatina	SLCO1B1 de función disminuida (c.521T>C rs4149056)^c^	Aumento de riesgo de miopatía comparado con función normal	Riesgo de miopatía en dosis >20 mg	Fuerte
	SLCO1B1 de función ineficaz (c.521T>C rs4149056)^c^	Aumento de riesgo de miopatía comparado con función normal y disminuida	Prescribir <20 mg. Si es necesario >20 mg considerar cambio de estatina o terapia combinada	Moderado
	ABCG2 de función normal (c.421 C/C rs2231142)^c^	Riesgo de miopatía típico	Prescribir según guías	Fuerte
	ABCG2 de función disminuida (c.421 C/A rs2231142)^c^	Aumento de riesgo de miopatía comparado con función normal	Prescribir según guías	Moderado
	ABCG2 de función ineficaz (c.421 A/A rs2231142)^c^	Aumento de riesgo de miopatía comparado con función normal y disminuida	Prescribir <20 mg. Si es necesario >20 mg considerar cambio de estatina o terapia combinada	Moderado
Simvastatina	SLCO1B1 de función disminuida (c.521T>C rs4149056)^c^	Aumento de riesgo de miopatía comparado con función normal	Prescribir estatina alternativa o limitar dosis a <20 mg	Fuerte
	SLCO1B1 de función ineficaz (c.521T>C rs4149056)^c^	Aumento de riesgo de miopatía comparado con función normal y disminuida	Prescribir estatina alternativa	Fuerte

^a^En todos los casos en los que hay un aumento de riesgo de miopatía es porque está asociado a un aumento de exposición del fármaco. ^b^Nomenclatura según CPIC. ^c^Variante genética y *reference SNP* (rs). Detallados en Tabla 3. Adaptado de [9].

En la Figura Suplementaria 1 se expone un ajuste de dosis para atorvastatina en función del fenotipo y en la Tabla Suplementaria 2 para simvastatina.

## Aspectos relevantes y perspectivas de futuro

En la actualidad diversos países han implementado la farmacogenética en rutina clínica [42, 49]. La mayoría de los modelos de implementación incluyen el análisis anticipado de un panel de 12 farmacogenes accionables (58 alelos) con un grado de evidencia alto (1A), y que permite establecer recomendaciones sobre el tratamiento de 57 fármacos [41].

Este desarrollo de la farmacogenética ha sido posible gracias a la labor de grupos de expertos que ha permitido superar los obstáculos en su implementación clínica [50] y que han aportado respuestas y las herramientas necesarias para: la identificación de los pares gene-fármacos con evidencia 1A; el desarrollo de guías farmacogenéticas con recomendaciones claras para la elección y ajuste del tratamiento; la estandarización metodológica y de la nomenclatura de los alelos; realizar el informe farmacogenético en sistemas electrónicos (compatibles con los utilizados en sanidad); avanzar en estadística genética o técnicas de *Machine Learning;* demostrar el coste-beneficio; la formación del personal sanitario y de la sociedad [9, 26, 49].

Estas primeras experiencias representan una gran oportunidad para estandarizar y mejorar los diferentes eslabones de todo el procedimiento (desde la solicitud del panel de farmacogenética anticipado hasta la elaboración del informe farmacogenético) y poder evaluar de forma adecuada la utilidad clínica de la farmacogenética como herramienta para seleccionar a los fármacos y dosis más adecuadas con el objetivo de reducir significativamente las RAM, y mejorar en la medida de lo posible la eficacia, de los tratamientos desde atención primaria [51, 52].

Un aspecto relevante en el análisis farmacogenético, además de la estandarización que mejorará la robustez de los resultados y facilitará su comparación entre diversos centros, es la necesidad de estar suscrito a Programas de Controles de Calidad Externos.

Centrándonos en la farmacogenética de las estatinas, se requieren estudios multicéntricos prospectivos en los que se evalúen de forma apropiada las interacciones medicamentosas, con repercusión en la actividad de los CYP y transportadores OATP1B1 y BCRP, que den lugar a un incremento en la exposición de las estatinas y al riego de padecer SAMS. Además, es necesario estudiar si la monitorización de las concentraciones de estos fármacos en plasma (fenotipo metabolizador) en combinación con el genotipo puede facilitar un ajuste de la dosis más personalizado.

Diversos estudios mostraron que los test farmacogenéticos en relación a las estatinas parecen ser costo-efectivos especialmente cuando estos genes se analizan en el contexto del panel farmacogenético de 12 genes, ya mencionado [42, 53].

El panel actual seleccionado para el test de farmacogenética anticipado irá mejorando con el tiempo y debe nutrirse desde la investigación con los nuevos hallazgos gen-fármaco de alta evidencia y con la inclusión del análisis de nuevos alelos o haplotipos identificados para pares de gen-fármaco ya establecidos. Referente a los genes de interés para las estatinas, a pesar de que las recomendaciones de las últimas guías se basan en reducir los SAMS, las investigaciones futuras podrán evaluar el impacto del test farmacogenético anticipado no sólo en el riesgo de SAMS sino también en la adherencia al tratamiento, en los niveles de cLDL y en el riesgo de enfermedades cardiovasculares [9].

Esta evolución en el conocimiento sobre el beneficio clínico de los test anticipados farmacogenéticos (y en su caso de la monitorización plasmática de los fármacos) debe tener difusión y es el principal motivo de la formación continuada del personal sanitario y de la sociedad.

En resumen, en esta nueva era de la implementación clínica de la medicina de precisión, el análisis farmacogenético anticipado de las estatinas conllevaria un claro beneficio clínico por su potencial para reducir de forma significativa la aparición de RAMs en los pacientes tratados. Por otra parte, es necesaria la estandarización de datos clínicos y metodologías analíticas para poder afinar las recomendaciones sobre el tratamiento personalizado con cada estatina.

## Supplementary Material

Supplementary Material

## Material Suplementario

Este artículo ofrece material suplementario (https://doi.org/10.1515/almed-2023-0064).
