# Supplementary material for: Influencia de la farmacogenética en la diversidad de respuesta a las estatinas asociada a las reacciones adversas
Source: Adv Lab Med. 2023 Oct 5;4(4):353–64. [Article in Spanish] doi: 10.1515/almed-2023-0064 (PMC10724860; doi:10.1515/almed-2023-0064)
Supplement: Supplementary file 1 — Supplementary Material [file j_almed-2023-0064_suppl_001.docx]

| **Tabla Suplementaria 1:** Transportador y complejo metabolizador implicado según estatina | |
| --- | --- |
| **Transportador** |  |
| ABCB1 | Atorvastatina, lovastatina, pravastatina, rosuvastatina, simvastatina, pitavastatina |
| ABCC2 | Atorvastatina, lovastatina, pravastatina, rosuvastatina, simvastatina, pitavastatina |
| ABCG2 | Atorvastatina, fluvastatina, pravastatina, rosuvastatina, pitavastatina |
| ABCB11 | Pravastatina, rosuvastatina |
| SLC15A1 | Fluvastatina |
| SLC22A6 | Pravastatina |
| SLC22A8 | Pravastatina |
| SLCO1B1 | Atorvastatina, fluvastatina, lovastatina, pravastatina, rosuvastatina, simvastatina |
| SLCO2B1 | Atorvastatina, fluvastatina, lovastatina, pravastatina, rosuvastatina, simvastatina |
| SLCO1B3 | Atorvastatina, fluvastatina, lovastatina, pravastatina, rosuvastatina, simvastatina, pitavastatina |
| SLCO10A1 | Atorvastatina, lovastatina, simvastatina |
| SLCO1A2 | Rosuvastatina |
| **Metabolismo** |  |
| CYP3A4 | Atorvastatina, lovastatina, simvastatina |
| CYP3A5 | Atorvastatina, lovastatina, simvastatina |
| CYP2C8 | Atorvastatina, fluvastatina, lovastatina, simvastatina |
| CYP2C9 | Atorvastatina, fluvastatina, lovastatina, rosuvastatina, simvastatina, |
| CYP2C19 | Atorvastatina, fluvastatina, lovastatina, simvastatina |
| CYP2D6 | Atorvastatina, lovastatina, simvastatina |
| UGT1A1 | Atorvastatina, fluvastatina, lovastatina, simvastatina |
| UGT1A3 | Atorvastatina, fluvastatina, lovastatina, simvastatina |
| UGT2B7 | Atorvastatina, lovastatina, simvastatina |
| Adaptado de [8,12]. | |

| **Tabla Suplementaria 2:** Recomendaciones de posología de simvastatina según el fenotipo SLCO1B1 | | | |
| --- | --- | --- | --- |
| **Fenotipo** | **Genotipo** | **Riesgo de miopatía** | **Recomendación** |
| Función eficaz, homocigoto | TT | Normal | Prescribir dosis deseada y ajustar según guías |
| Función intermedia, heterocigoto | TC (c.521T>C rs4149056)^a^ | Intermedio | Prescribir dosis menor en comparación con riesgo normal o considerar una estatina alternativa |
| Función ineficaz, homocigoto | CC (c.521T>C rs4149056)^a^ | Alto | Prescribir una dosis menor en comparación con riesgo intermedio y normal o considerar una estatina alternativa |
| ^a^ Variante genética y *reference SNP* (rs) presente en *SLCO1B1*5* y *SLCO1B1*15*  Adaptado de [8]. | | | |

**Atorvastatina**

**SLCO1B1**

**Función ineficaz**

**SLCO1B1**

**Función disminuida**

10-20mg

40 mg

80 mg

10-20mg

**40-80 mg**

Riesgo moderado SAMS

Riesgo alto SAMS

Riesgo bajo SAMS

Riesgo alto SAMS

Riesgo bajo SAMS

**Cambio de dosis o de estatina**

**Figura Suplementaria 1.** Ajuste de dosis de atorvastatina según el fenotipo. Adaptado de [9].
